# Subcellular organization of UBE3A in human cerebral cortex

**DOI:** 10.1186/s13229-018-0238-0

**Published:** 2018-10-19

**Authors:** Alain C. Burette, Matthew C. Judson, Alissa N. Li, Edward F. Chang, William W. Seeley, Benjamin D. Philpot, Richard J. Weinberg

**Affiliations:** 1 Department of Cell Biology and Physiology, University of North Carolina at Chapel Hill, 314 Taylor Hall, Campus, Box 7545, Chapel Hill, NC 27599-7545 USA; 2 Department of Cell Biology and Physiology and Neuroscience Center, University of North Carolina, Chapel Hill, NC 27599 USA; 3 Department of Neurology, University of California, San Francisco, CA USA; 4 Department of Pathology, UCSF Weill Institute for Neurosciences, University of California, San Francisco, CA USA; 5 Department of Neurological Surgery, University of California, 505 Parnassus Avenue, Box 0112, San Francisco, CA 94143-0112 USA; 6 Neuroscience Curriculum, Carolina Institute for Developmental Disabilities, University of North Carolina at Chapel Hill, Chapel Hill, NC 27599 USA

**Keywords:** Angelman syndrome, E6-AP, Axon terminal, Mitochondria, Euchromatin

## Abstract

**Background:**

Loss of UBE3A causes Angelman syndrome, whereas excess UBE3A activity appears to increase the risk for autism. Despite this powerful association with neurodevelopmental disorders, there is still much to be learned about UBE3A, including its cellular and subcellular organization in the human brain. The issue is important, since UBE3A’s localization is integral to its function.

**Methods:**

We used light and electron microscopic immunohistochemistry to study the cellular and subcellular distribution of UBE3A in the adult human cerebral cortex. Experiments were performed on multiple tissue sources, but our results focused on optimally preserved material, using surgically resected human temporal cortex of high ultrastructural quality from nine individuals.

**Results:**

We demonstrate that UBE3A is expressed in both glutamatergic and GABAergic neurons, and to a lesser extent in glial cells. We find that UBE3A in neurons has a non-uniform subcellular distribution. In somata, UBE3A preferentially concentrates in euchromatin-rich domains within the nucleus. Electron microscopy reveals that labeling concentrates in the head and neck of dendritic spines and is excluded from the PSD. Strongest labeling within the neuropil was found in axon terminals.

**Conclusions:**

By highlighting the subcellular compartments in which UBE3A is likely to function in the human neocortex, our data provide insight into the diverse functional capacities of this E3 ligase. These anatomical data may help to elucidate the role of UBE3A in Angelman syndrome and autism spectrum disorder.

## Background

Protein degradation through the ubiquitin-proteasome pathway (UPP) is important for a wide range of functions in the nervous system [1, 2]. In UPP processing, the protein substrate is marked by covalent attachment of the small protein ubiquitin for subsequent degradation by the proteasome, a specialized proteolytic complex. The process starts with the activation of ubiquitin by E1 ubiquitin ligase enzymes [3, 4]. E2 enzymes then transfer the activated ubiquitin to E3 ubiquitin ligases, which attach the ubiquitin to the substrate. The E3 ligases determine the substrate specificity of ubiquitination.

Two major classes of E3s have been defined: HECT (homologous to E6-AP carboxyl-terminus) domain E3s and RING finger E3s. UBE3A, the first E3 discovered in the HECT class [5], is of special interest, because failure to maintain appropriate levels of UBE3A in the brain is directly linked to severe clinical disorders. The loss of UBE3A function causes Angelman syndrome (AS) [6, 7], characterized by profound intellectual and developmental disabilities [8]. On the other hand, increased UBE3A activity (via gene duplication or gain-of-function mutation) is implicated in several forms of autism [9–15], implying that UBE3A must be expressed at precise levels to allow proper neuronal function.

Despite its clinical importance, our current understanding of UBE3A’s function in the brain remains limited. UBE3A has been implicated in regulation of Golgi acidification [16] and mitochondrial function [17–19], as well as synaptic transmission and plasticity [20–26]. Several potential interactors and substrates for its ubiquitin ligase activity have been identified, including the RhoA guanine nucleotide exchange factors Pbl/ECT2 and Ephexin5 [24, 27, 28], RAD23A, (an enzyme involved in excision repair of damaged DNA [29]), and the estrogen receptor [30]. UBE3A has also been shown to regulate the levels of Cbln1 in excitatory neurons, which in turn can influence sociability [26]. Finally, UBE3A can interact directly with the proteasome to influence its degradative activity [15, 31–35], suggesting potentially wide-ranging roles for UBE3A in cellular functions that remain to be fully defined.

Crucial for the elucidation of UBE3A’s functions is to determine where the protein actually lies within the cell. Previous immunohistochemical studies in rodents found a broad distribution of UBE3A, but emphasized its nuclear expression in mature neurons, consistent with a proposed role for UBE3A in the regulation of transcription [26, 36–38]. However, patterns of UBE3A expression in humans may not be fully conserved, considering the 100 million years of divergent evolution between rodents and primates. Unique patterns of UBE3A expression within the cerebral cortex could have important functional implications, especially given the cognitive manifestations of both AS and autism. Here, we explore this intriguing possibility by investigating the subcellular distribution of UBE3A in the human temporal cortex.

## Methods

### Human brain tissue

All procedures regarding human tissue were performed with the approval of the UCSF Committee on Human Research and the University of Maryland Institutional Review Board. Nine small blocks from postmortem temporal cortex were obtained from the UCSF Neurodegenerative Disease Brain Bank (NDBB) and the NIH NeuroBioBank, including autopsy materials from all three adults with AS currently available for study. Details are listed in Table 1. Interpretability of immunohistochemical results depends on the quality of the tissue. To assess structural preservation, we used transmission electron microscopy of fixed samples embedded in epoxy plastic.

**Table 1:** Autopsy cases included in this study

Repository	Case ID	Sex	Age (years)	Clinical diagnosis	Time to fixation (hours)	RIN
UCSF NDBB	P2321.10	M	77	MCI	4.9	3–3.6
UCSF NDBB	P2394.12	M	76	Control	8.2	5.2–6.1
UCSF NDBB	P2698	M	81	MCI	10	–
Maryland	5363	F	28	Control	11	–
Maryland	5659	M	17	Control	10	6.8
Maryland	5758	F	43	Control	18–23	–
Maryland	1494	F	43	AS	21	–
Maryland	5743	M	19	AS	18–23	–
Maryland	3854	F	21	AS	21	–

**Fig. 1:**
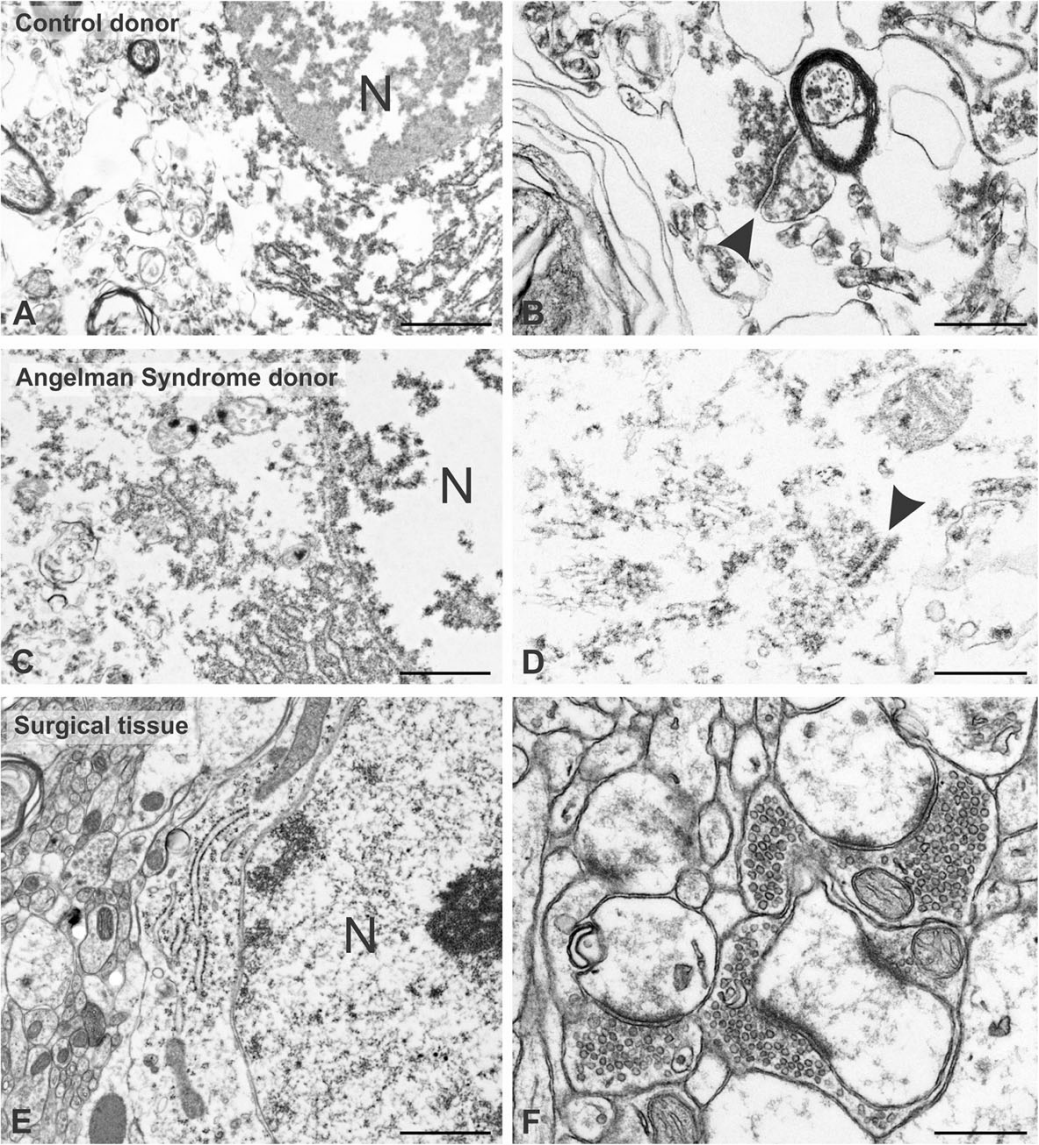
Ultrastructural integrity of human tissue samples from different sources. Ultrastructural integrity is low in postmortem material from a control donor, though major organelles such as nuclei (N) remain identifiable (low-magnification view, **a**). At high magnification, synapses (arrowhead) are damaged but clearly defined (**b**). Material from an Angelman syndrome donor is virtually uninterpretable (reflecting the practical difficulty of obtaining and curating such material), though damaged mitochondria and traces of nuclear structure remain (N in panel **c**); hints of synaptic specializations may be identified even in this material (arrowhead, **d**). In contrast, tissue from biopsy specimens obtained during surgeries for epilepsy shows very good preservation, as documented by features including intact mitochondria, well-aligned rough endoplasmic reticulum, and intact chromatin (**e**). The well-preserved neuropil (**f**) contrasts with that of the autopsy specimens above. Tissue from case no. 5758 for **a** and **b**, 1494 for **c** and **d**, and Q1012 for **e** and **f**. Scale bar = 1 μm in **a**, **c**, and **e** and 500 nm in **b**, **d**, and **f**

The ultrastructural integrity of all postmortem tissue was seriously compromised. In the best preserved samples, most subcellular structures were identifiable, but the tissue contained abnormal holes, neurons were vesiculated, cytoplasm was disrupted, nucleoplasm appeared abnormally condensed, membranes were distorted, and mitochondria were swollen (Fig. 1a, b). The overall impression was of substantial deterioration of the material surrounding a “skeleton” of residual organization. Notwithstanding the suboptimal integrity of this material, it was far superior to that of the available postmortem samples from persons with AS (Fig. 1c, d). This material was markedly degraded, with severe disruption and a global lack of ultrastructural integrity. A few elements remained identifiable, including many synapses, but these resembled biochemically isolated preparations; synaptic membranes and synaptic vesicles were typically lost or distorted. We concluded that despite their inherent clinical importance, the available AS samples were unsuitable for micro-anatomical study, while autopsy material from neurotypical controls was acceptable for specific purposes if results were interpreted with caution. Unexpectedly, the structural preservation and RIN score (RNA integrity number) were not always correlated (Table 1), further emphasizing the value of ultrastructural assessment.

Choosing to focus on well preserved material, we obtained 9 surgical biopsies. Because of limited tissue availability, all nine were from patients undergoing surgery to remove epileptic foci in the hippocampus (Table 2). The temporal cortex from these patients was intact, displaying normal activity in intraoperative electrocorticography, and removed only to gain access to the pathological hippocampal tissue beneath it. Small pieces of the neocortex overlying the epileptic focus were immersion-fixed for 24 h at 4 °C in 1–4% paraformaldehyde (PFA)/1% glutaraldehyde in phosphate buffer (PB), 2% PFA/2–4% glutaraldehyde in 0.1 M sodium cacodylate + 0.1% CaCl_2_ and 2.5% DMSO, or 4% PFA in 0.1 M sodium cacodylate + 0.1% CaCl_2_ and 2.5% DMSO. Following fixation, the samples were stored in PO_4_-buffered saline (PBS) at 4 °C. Sections were cut at 50 μm on a Vibratome and collected in cold PB.

**Table 2:** Biopsy cases included in this study

Case no.	Sex	Age (years)	Clinical diagnosis	Time to fixation (minutes)
Q1010	M	30	Epilepsy	15
Q1011	M	44	Epilepsy	6
Q1012	M	58	Epilepsy	10
Q1013	F	38	Epilepsy	6
Q1014	F	48	Epilepsy	10
Q1015	F	66	Epilepsy	7
Q1017	M	46	Epilepsy	7
Q1018	F	20	Epilepsy	5
Q1019	F	51	Epilepsy	4

In contrast to the postmortem tissue, the ultrastructural integrity of the biopsy samples was generally good, and in many cases, comparable to that obtained from perfusion-fixed mice (Fig. 1e, f). Since the biopsies were from patients undergoing surgery to remove epileptic foci, we took the added precaution of staining each sample for parvalbumin, GABA, and glial fibrillary acidic protein (GFAP), three markers which may undergo pathological changes in epileptic tissues; we restricted our analysis to regions from each biopsy that appeared normal, as assessed by comparison with immunostaining in perfusion-fixed rodents.

### Animals

All procedures related to the care and treatment of animals were in accordance with institutional and NIH guidelines, and all animal use protocols were reviewed and approved by the UNC Institutional Animal Care and Use Committee. AS model mice (*Ube3a*^*m−/p+*^), which maternally inherit the *Ube3a* knock-out allele, were generated by mating wild-type (*Ube3a*^*m+/p+*^) male mice to female mice with paternal inheritance of the *Ube3a* knock-out allele (*Ube3a*^*m+/p–*^), which themselves are phenotypically normal [39, 40]. After inducing deep anesthesia with sodium pentobarbital (80 mg/kg, i.p.), adult (P60–P100) male mice were intracardially perfused with 50 ml of 4% freshly depolymerized paraformaldehyde in phosphate buffer (PB, 0.1 M, pH 7.4), then post-fixed overnight at 4 °C in the same buffer. To mimic the biopsy procedure in human patients, we anesthetized mice with pentobarbital (80 mg/kg) and quickly removed the brain. One hemisphere was immediately submerged in fixative, while the other half was exposed to room air for 15 min prior to immersion in fixative. After 24 h in the fixative, the brains were rinsed in PB, sectioned at 50 or 100 μm on a Vibratome, and collected in cold PB.

### Antibodies

To identify UBE3A, we used a mouse monoclonal antibody (3E5, Sigma-Aldrich, cat. no. SAB1404508, RRID:AB_10740376) raised against a peptide corresponding to mouse UBE3A isoforms 1 and 3 (a.a. 315–415) and isoform 2 (a.a. 336–436). Importantly, this immunogen shares 100% sequence identity with human UBE3A isoforms 1 (a.a. 318–417), 2 (a.a. 341–441), and 3 (a.a. 338–437). We used this antibody because we had previously verified its specificity against AS model and UBE3A KO mice [41].

To identify GABA, we used a rabbit polyclonal antibody (Sigma, St. Louis, MO; A2052, lot 052K4827) developed using GABA-BSA as the immunogen. The antibody was affinity-purified using the immunogen. It showed positive binding with GABA and GABA-KLH in a dot blot assay and negative binding with bovine serum albumin (BSA) (manufacturer’s technical information).

To identify GFAP, we used a rabbit polyclonal anti-GFAP (Chemicon, AB5804) developed against purified bovine GFAP. This antibody recognizes a 51 kDa band on immunoblot of rat spinal cord.

To identify Oligodendrocyte Transcription Factor 2 (Olig2), we used a rabbit polyclonal anti-Olig2 (Millipore, AB9610) developed using a recombinant mouse Olig-2. This antibody specifically labels cells of oligodendrocyte lineage [42], consistent with our own observations [43].

### Light microscopy

Free-floating sections were treated for 30 min in 1% sodium borohydride, followed by 30 min with 3% H_2_O_2_ in phosphate-buffered saline (PBS, 0.1 M, pH 7.4). After preincubation in 10% normal donkey serum (to block secondary antibody binding sites), sections were incubated in UBE3A (1:500 to 1:5000) overnight on a shaker at room temperature.

For immunoperoxidase microscopy, sections were then incubated overnight on a shaker at room temperature in biotinylated secondary antibody (1:200; Jackson ImmunoResearch, West Grove, PA) and for 4 h in ExtrAvidin-peroxidase complex (1:5000; Sigma, St. Louis, MO); peroxidase was histochemically visualized with diaminobenzidine. Processed sections were mounted on gelatin-coated slides, air dried, and cleared with xylene before being coverslipped with D.P.X. Mountant (BDH Chemicals, Poole, England).

For immunofluorescence, antigenic sites were visualized with donkey IgG conjugated to DyLight 549 (1:200, Jackson ImmunoResearch; West Grove, PA). For double labeling, the second primary antibody (1:10,000 GABA, 1:1000 Olig2, or 1:2000 GFAP) was applied overnight and visualized by a secondary antibody conjugated to Alexa 488 (1:200, Invitrogen). Some sections were then counterstained with DAPI (1:10,000, Sigma-Aldrich, cat. no. D9542) to visualize nuclei, and with NeuroTrace 640–660 (1:1000, Invitrogen, Thermo Fisher Scientific, Rockford, IL; cat. no. N21483) to visualize neuronal somata. Sections were examined with a Zeiss LSM 880 confocal microscope with Airyscan.

### Electron microscopy

To assess structural preservation, sections were processed for reduced osmium according to the Knott protocol [44, 45]. Briefly, sections were washed in cacodylate buffer (0.1 M, pH 7.4) and post-fixed for 40 min in 1.5% potassium ferrocyanide and 1% osmium tetroxide, followed by 1 h in 1% osmium tetroxide alone, rinsed in water, then incubated 40 min in 1% uranyl acetate in water, rinsed in water, dehydrated in an ethanol series, and infiltrated and embedded in resin (Spurr’s low viscosity epoxy with ERL-4221, Electron Microscopy Sciences, Hatfield, PA; cat. no. 14300).

For immunohistochemistry, sections were treated with 1% sodium borohydride/PBS, followed by 3% H_2_O_2_/PBS, then 0.025% Triton X-100/PBS, then blocked with 10% normal goat serum (NGS), and incubated overnight in primary antibody (UBE3A, 1:200 to 1:1000). Sections were rinsed, and blocked again in 2% NGS, then incubated overnight with biotinylated goat-anti mouse IgG (1:2000, Vector Laboratories, Burlingame, CA; cat. no. BA-9200), rinsed, and incubated for 2 h in 1.4 nm gold particles conjugated to streptavidin (1:100; Nanoprobes, Yaphank, NY; cat. no. 2016). After rinsing, binding was stabilized by treating 30 min in 2% glutaraldehyde/PBS; sections were washed again and transitioned into 0.05 M sodium acetate (to remove phosphate and chloride ions), followed by silver enhancement with HQ Silver (Nanoprobes, Yaphank, NY; cat. no. 2012). After rinsing in sodium acetate and transitioning to PB, sections were post-fixed in 0.5% osmium tetroxide in 0.1 M PB for 45 min, rinsed and transitioned to 0.1 M maleate buffer, pH 6, and then incubated for 45 min with 1% uranyl acetate in maleate buffer (0.1 M, pH 6.0).

After processing, sections were dehydrated in a graded ethanol series and infiltrated with a low viscosity epoxy resin (Spurr’s with ERL-4221, Electron Microscopy Sciences, Hatfield, PA) and flat-mounted between sheets of ACLAR® fluoropolymer (Electron Microscopy Sciences, Hatfield, PA; cat. no. 50425) within glass slides. Sixty-nanometer sections were cut and mounted on 200 mesh copper grids. For double labeling with GABA, nickel grids were pretreated 15 min at 60 °C in 0.01 M citrate buffer, pH 6, rinsed in water, blocked in 1% BSA in Tris-buffered saline, pH 7.6, with 0.005% Tergitol NP-10, and incubated overnight at 21–24 °C room temperature with the primary antibody (GABA, 1:10,000 to 1:50,000). Grids were rinsed, blocked in 1% normal goat serum in TBS, pH 8.2, with Tergitol NP-10, and incubated in secondary antibodies for 2 h (goat antibody to rabbit IgG conjugated to 20 nm gold particles, BBI, UK, cat. no. EM.GAR20). Grids were then rinsed in buffer, then water, and counterstained with 1% uranyl acetate in water, followed by Sato’s lead. Electron microscopy was performed with a Philips Tecnai transmission electron microscope.

### Controls

**Fig. 2:**
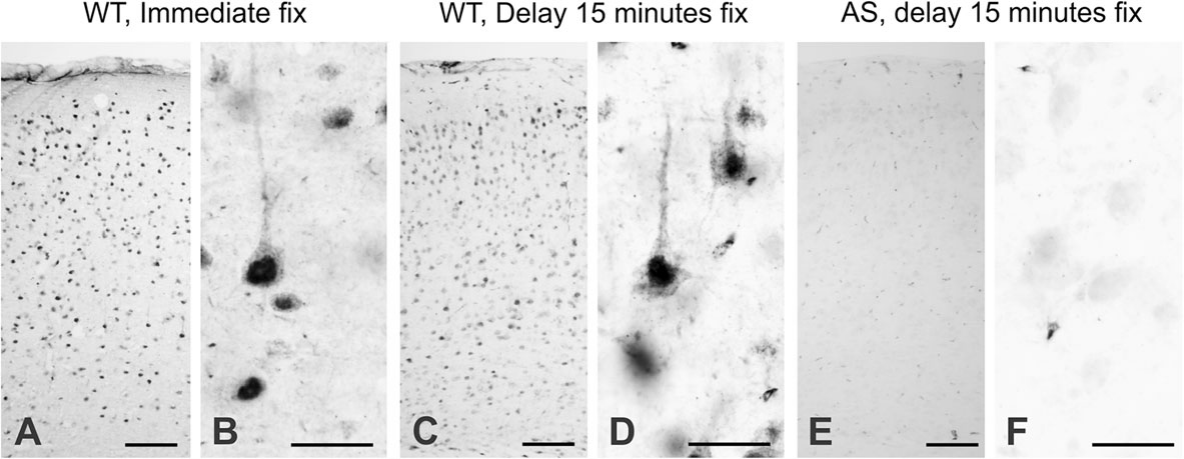
Immunohistochemistry for UBE3A in mouse cerebral cortex following different methods of fixation. UBE3A staining in tissue fixed by perfusion (**a**, **b**). **c**, **d** Immersion fixation of mouse cortex after a delay of 15 min (similar to the fixation for our surgical material) shows only modest differences, including a subtle condensation of immunopositive cytoplasm. In both cases, UBE3A is expressed through all layers of the neocortex, with strong nuclear staining and weaker staining in somata and dendrites. UBE3A staining is nearly absent in the brain of an AS model mouse fixed by immersion after a delay of 15 min (**e**, **f**), further confirming specificity of the UBE3A antibody in brain tissue harvested under conditions similar to surgical biopsy. Scale bar = 100 μm in **a**, **c**, and **e** and 25 μm in **b**, **d**, and **f**

Biopsy specimens are subject to an unavoidable delay (for our material, 5–15 min, Table 2) prior to immersion in the fixative. Delayed fixation could in principle lead to misleading artifacts in immunolocalization, particularly for a protein involved in proteasomal degradation like UBE3A. Accordingly, we verified that similarly delayed immersion fixation in mice does not significantly affect UBE3A distribution or cause other unforeseen issues. We compared the overall distribution of UBE3A in mouse brains fixed by perfusion, immediate immersion fixation, and immersion fixation after a delay of 15 min (Fig. 2). We found that UBE3A staining was similar in all three cases: it was expressed throughout all layers of the neocortex, with strong nuclear staining and weaker staining in somata and dendrites. Vascular artifacts and subtle indications of cytoplasmic condensation after delayed fixation did not significantly affect the pattern of immunostaining.

To verify antibody specificity, we performed the same procedures on AS model mice (maternal UBE3A knock-out); only a very weak background staining could be detected in these mice (Fig. 2e, f). We conclude that a 15-min delay in fixation does not significantly alter the distribution of UBE3A.

### Image analysis

To compare the level of nuclear UBE3A between excitatory neurons and GABAergic neurons, we acquired 58 random fields of human cortical layers II/III (100 μm × 100 μm, from Q1010, Q1011, and Q1012) from material quadruple-stained for UBE3A, GABA, NeuroTrace, and DAPI. To minimize artifacts arising from limited penetration, confocal images were acquired just below the section surface. For each neuron, the intensity of UBE3A staining in the nucleus (defined by DAPI) was computed using FIJI [46]. To control for possible local variations of immunostaining intensity arising from immersion fixation, we computed the ratio of staining in GABA-positive and GABA-negative neurons from each field individually, reasoning that NeuroTrace-positive neurons that were immunonegative for GABA were very likely to be excitatory.

To examine the quantitative relationship between staining for UBE3A and DAPI, we performed intensity correlation analysis as described by Li et al. [47], using FIJI. The analysis was performed on 92 random nuclei from human cortical layers II/III (from Q1010, Q1011, and Q1012, which yielded particularly robust immunostaining for UBE3A). The imaging was done with the Airyscan mode.

## Results

**Fig. 3:**
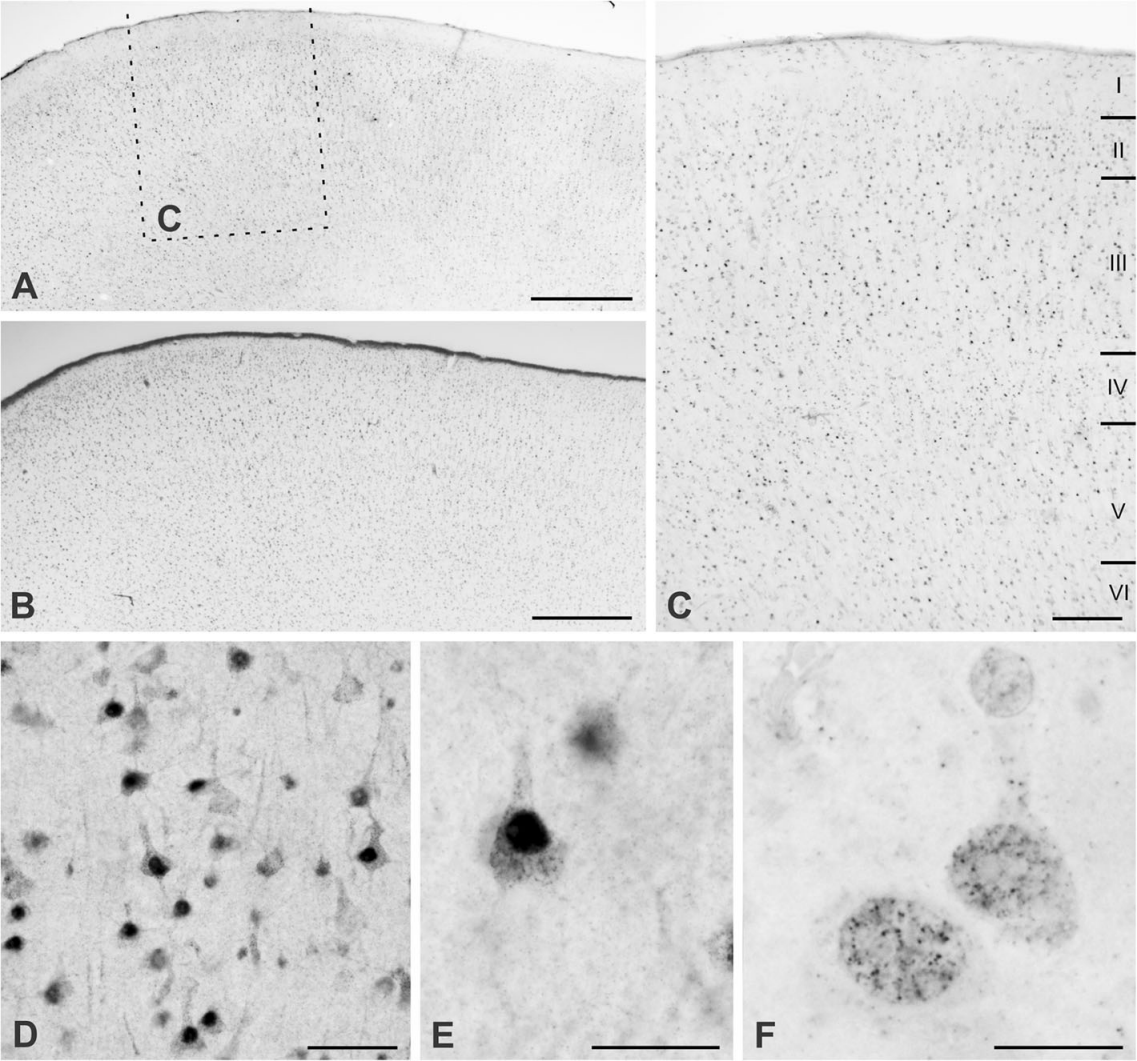
UBE3A expression in temporal cortex from a human biopsy specimen. **a** Immunoperoxidase staining for UBE3A. As can be seen by comparison with adjacent Nissl-stained section (**b**), the large majority of neurons are immunopositive. **c** Enlargement of boxed area from **a**; UBE3A is expressed throughout all layers of temporal cortex. **d**, **e** Higher magnification micrographs show UBE3A concentrating in nuclei. Antigen is also visible in somata and neuropil, but at lower levels. **f** High magnification micrograph of a 250-nm plastic section shows discrete grains of immunoperoxidase reaction product scattered through most of the nucleus, but seemingly excluded from certain regions. Tissue from case no. Q1019. Scale bar = 1 mm in **a** and **b**, 250 μm in **c**, 50 μm in **d**, 25 μm in **e**, and 10 μm in **f**

The overall pattern of staining was consistent across biopsy samples from all 9 subjects. UBE3A was found in neurons throughout the depth of cerebral cortex (Fig. 3a–c). Staining within neurons concentrated in the nucleus, but could also be seen in somata and proximal dendrites, and punctate staining was observed in neuropil (Fig. 3d, e). The apparent lack of nuclear staining in some neurons was interpreted as an artifact arising from limited antibody penetration, since it was not seen in neurons whose nucleus lay at the section edge. Examination of thin (< 300 nm) sections showed that much of the nuclear staining was organized into discrete tiny aggregates of DAB reaction product distributed through the nucleus (Fig. 3f).

**Fig. 4:**
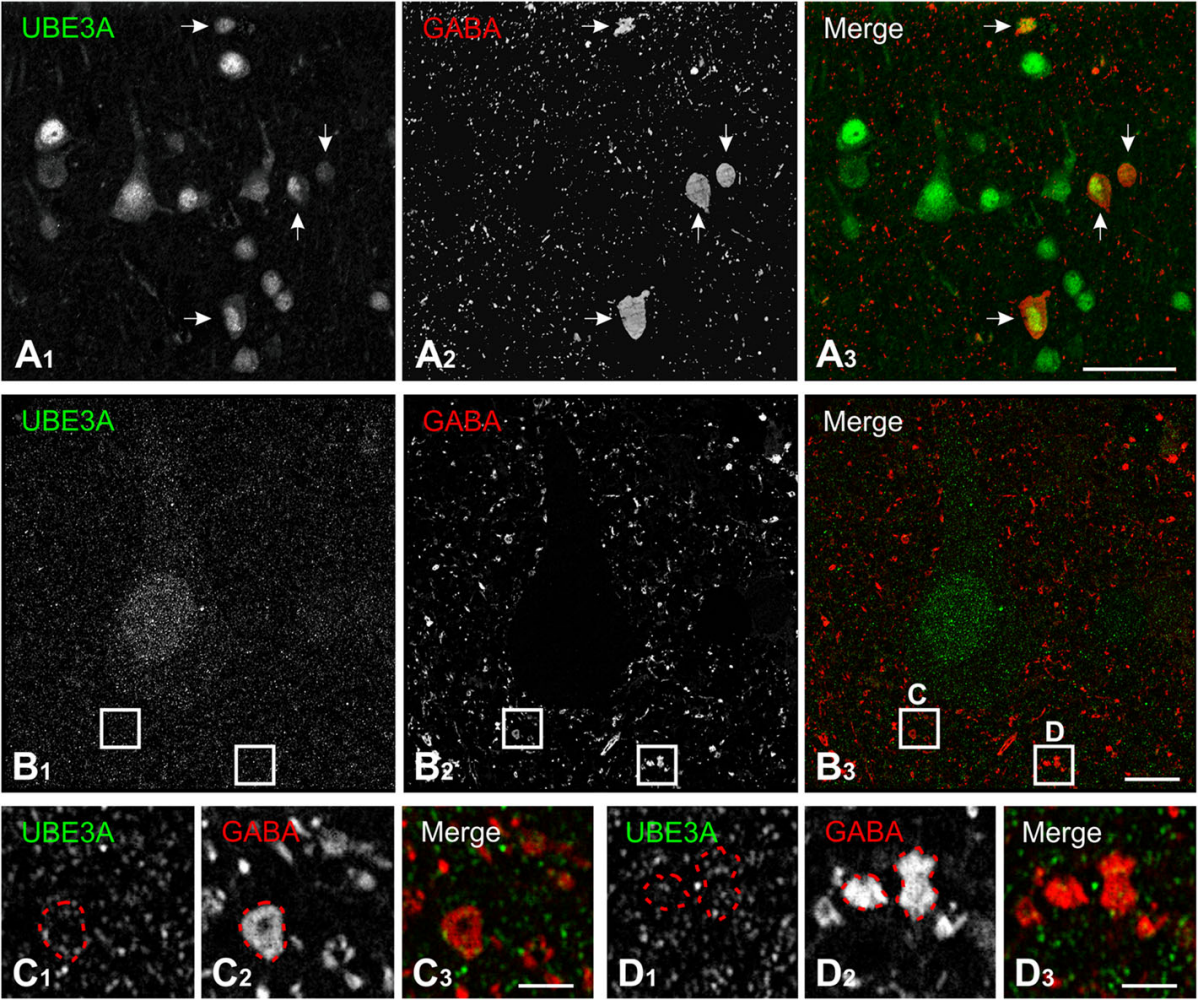
UBE3A expression in GABAergic neurons in temporal cortex from a human biopsy specimen. **a** Double labeling reveals UBE3A in the nuclei and somata of GABAergic neurons (arrows). **b** A pyramidal neuron immunopositive for UBE3A is surrounded by numerous GABA-positive structures likely to represent cross sections of dendrites and axon terminals. Enlargements in **c** and **d** show the presence of UBE3A in some of these small GABA-positive structures. Tissue from case no. Q1010. Scale bar = 50 μm in **a**, 10 μm in **b**, and 2 μm in **c** and **d**

GABAergic deficits have been implicated in the pathogenesis of neocortical hyperexcitability and epilepsy in AS model mice [25, 48], and GABA transporter-1 has been proposed as a potential UBE3A substrate [49]. To determine whether UBE3A is expressed in inhibitory neurons of the human neocortex, as it is in mice, we performed double labeling with GABA (Fig. 4). We found that GABAergic neurons, like pyramidal neurons, express higher levels of UBE3A in their nuclei than in their somata. However, quantitative analysis of biopsy material from three subjects revealed that the concentration of nuclear UBE3A is ~ 40% (38 ± 6.4%, *n* = 51) higher in pyramidal neurons than in GABAegic neurons. In addition, colocalization was observed in fine processes and puncta distributed through the neuropil (Fig. 4c, d).

**Fig. 5:**
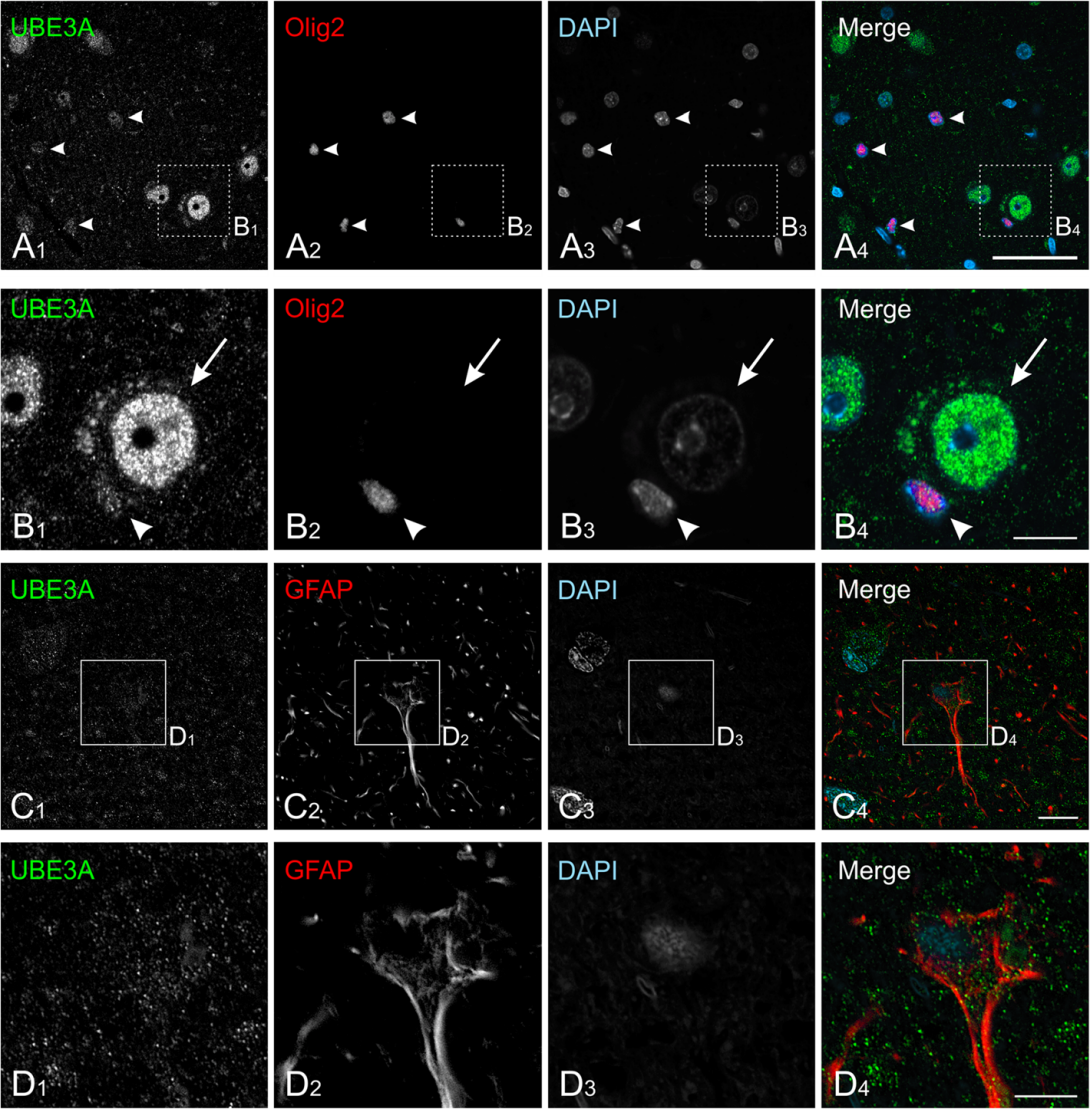
UBE3A expression in neuroglia in temporal cortex from human biopsy specimen. **a**, **b** Double labeling reveals UBE3A in the nuclei and somata of Olig2-positive glia (oligodendrocytes, arrowheads). However, UBE3A staining in Olig2-positive glia (**b**, arrowhead) is appears weaker than in neurons (**b**, arrow). **c** Double labeling reveals UBE3A in the nuclei and somata in GFAP-positive cells (astrocytes). UBE3A in GFAP-positive cells is at lower levels than in neurons and Olig2-positive glia. Tissue from case no. Q1010. Scale bar = 50 μm in **a**, 10 μm in **b** and **c**, and 5 μm in **d**

In mice, UBE3A is expressed not only in neurons but also neuroglia. To evaluate its distribution in non-neuronal cells in the human temporal cortex, we performed double staining with Olig2, to identify oligodendrocytes (Fig. 5a, b). Indeed Olig2-positive somata were consistently immunopositive for UBE3A, though to a lesser degree than seen in neurons. We also performed double staining with GFAP to identify astrocytes, finding that astrocytes also expressed UBE3A, though generally at lower levels than either neurons or oligodendrocytes (Fig. 5c, d). These patterns were reminiscent of UBE3A staining of neuroglia in the mouse brain [43].

**Fig. 6:**
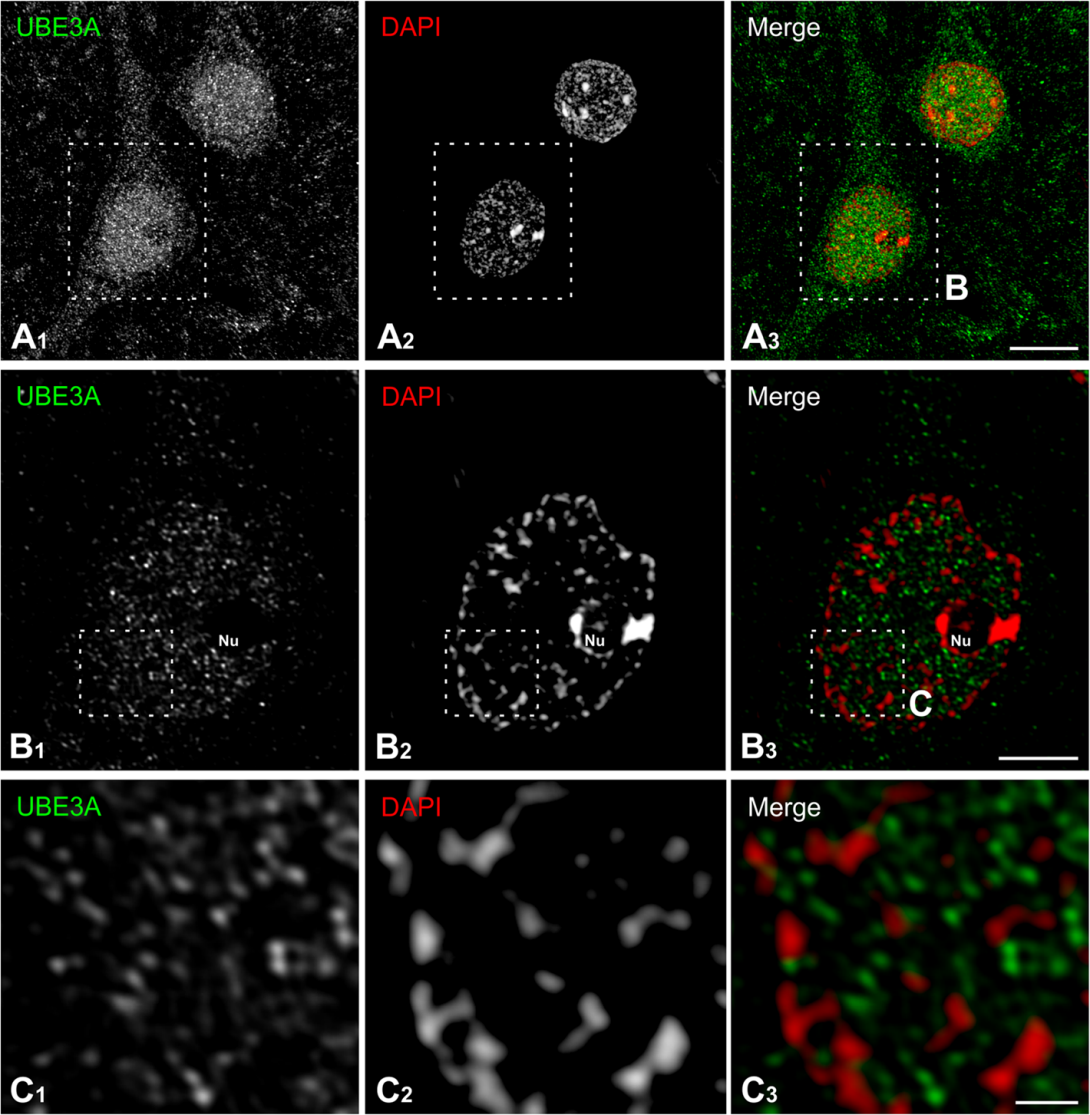
High magnification immunofluorescence, showing UBE3A (green) in two pyramidal neurons in layer V in temporal cortex from human biopsy specimen (**a**). Sections were counterstained with DAPI (red) to visualize nuclei. UBE3A staining is organized into small puncta that concentrate in neuronal nuclei. Small UBE3A-positive puncta are visible in somata and proximal dendrites, and throughout the neuropil. Successive enlargements (**b**, **c**) show that UBE3A puncta are excluded from the nucleolus (Nu) and from DAPI “hotspots.” Scale bar = 10 μm in **a**, 5 μm in **b**, and 1 μm in **c**. Tissue from case no. Q1019

**Fig. 7:**
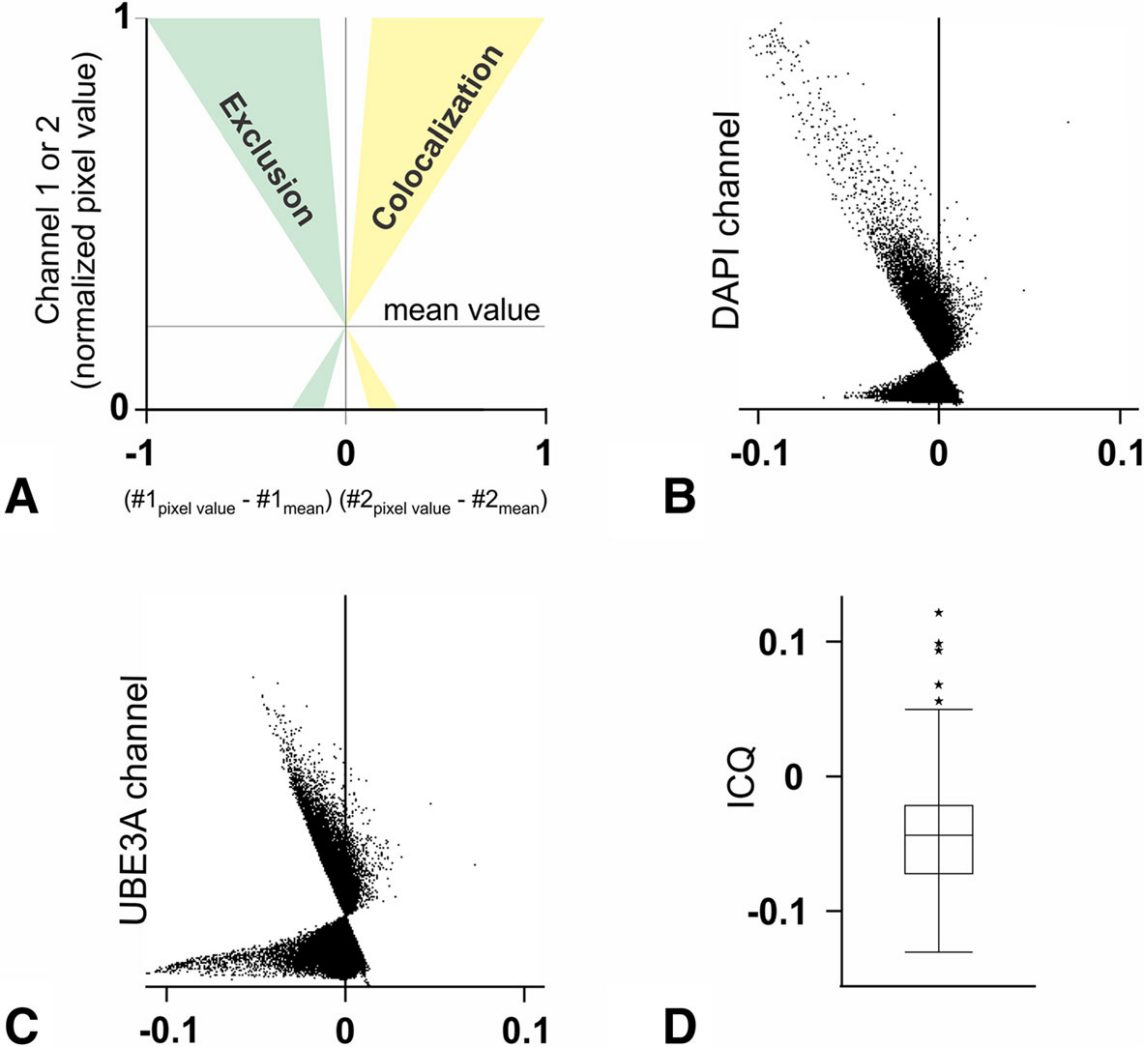
Quantification of the reciprocal localization of UBE3A and DAPI in human neuronal nuclei. To analyze the relationship between UBE3A and DAPI in neuronal nuclei, we used the intensity correlation analysis approach of Li et al. [47]. Analyses are presented as intensity correlation plots: the *x* value, (channel 1 pixel value–channel 1 mean value) × (channel 2 value–channel 2 mean value), reflect the covariance of both channels, and the *y* value reflects the intensity of channels 1 or 2. Pixels with values situated left of the *x* = 0 line do not colocalize or have inversely correlated intensities, whereas pixels situated on the right side colocalize (see diagram in **a**). Scatterplot in **b** and **c** corresponds to the nuclear region of the sections used for illustration in Fig. 6b (Scatterplots are from raw confocal images, while contrast and brightness were adjusted in the micrographs). Panel **c** shows DAPI with respect to UBE3A, and **d** shows UBE3A with respect to DAPI. Both plots are skewed toward negative values, implying that UBE3A and DAPI pixel intensity co-varies in opposite directions. **d** Box and whiskers plot of intensity correlation quotient (ICQ, [47]) from 92 nuclei from layers II–III. ICQ values ~ 0 imply random staining, 0 > ICQ ≥ − 0.5 indicate segregated (negatively correlated) staining, and 0 < ICQ ≤ + 0.5 indicate dependent (positively correlated) staining. We found an average ICQ of − 0.04 ± 0.005, showing that UBE3A and DAPI staining negatively co-vary

To get a better understanding of the subcellular organization of UBE3A, we performed high-resolution immunofluorescence microscopy. In images prepared using an Airyscan confocal microscope (Fig. 6), UBE3A staining appeared as discrete puncta whose density was high in the nucleus and also present (though at lower densities) in somata and proximal dendrites; scattered puncta of immunolabel were seen in neuropil (Fig. 6a). In the nucleus, UBE3A puncta were interspersed with DAPI “hotspots,” but did not overlap with them (Fig. 6b, c). Likewise, UBE3A staining seemed to be excluded from nucleoli (*Nu*, Fig. 6b). To more directly test the relationship between UBE3A and DNA condensation, we computed the intensity correlation quotient [47] (ICQ, computed such that − 0.5 represents perfect negative covariance and 0.5 represents perfect positive covariance between two antigens) for 92 nuclei from 3 humans Q1010, Q1011, and Q1012 (Fig. 7). We found an average ICQ of − 0.04 ± 0.005, showing that UBE3A and DAPI staining negatively co-vary, confirming our qualitative impression that UBE3A is excluded from regions of condensed DNA.

**Fig. 8:**
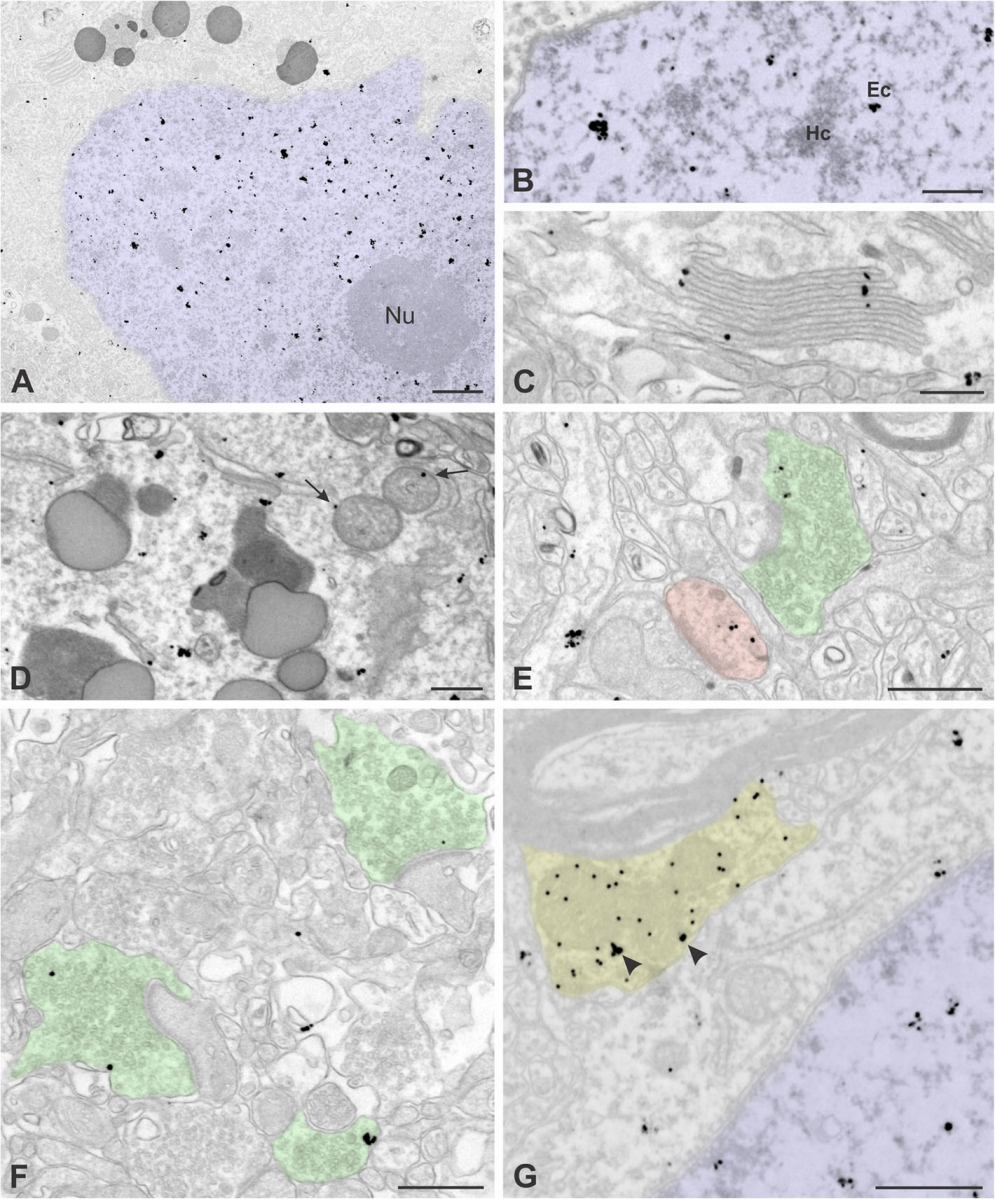
Pre-embedding immunogold labeling for UBE3A in layer II/III lateral temporal cortex from human biopsy specimen. **a**, **b** UBE3A labeling in nucleus (colorized in blue); labeling is over euchromatin (Ec) domains, but is not associated with heterochromatin (Hc) or nucleoli (Nu). **c** UBE3A labeling associated with endomembranes of the Golgi apparatus. **d** UBE3A labeling in cytoplasm; arrows point to label associated with mitochondria. **e**, **f** UBE3A labeling in presynaptic terminals (colorized in green) and a dendritic spine (colorized in red). **g** A GABAegic terminal (colorized in yellow) labeled for UBE3A (pre-embedding, irregular 30–50 nm particles of silver-intensified immunogold, arrowheads) and GABA (post-embedding, 20 nm gold). Tissue from case no. Q1010 in **a**, **c**, **d**, **e**, and **f** and Q1019 in **b** and **g**. Scale bar = 1 μm in **a** and **b**, 250 nm in **c** and **g**, and 500 nm in **d**–**f**

We used electron microscopy to examine the subcellular organization of UBE3A with higher precision, using pre-embedding immunogold labeling followed by silver intensification (Fig. 8). We focused our attention on layers II–III as we had done previously for mouse [41]. Labeling was prominent in nuclei but was also present in somatic cytoplasm and neuropil, echoing the results from light microscopy (Fig. 6). In the nucleus, UBE3A labeling was largely restricted to euchromatin domains; very little labeling was found over electron-dense heterochromatin zones (Fig. 8a, b), as was predicted by our ICQ analysis of UBE3A/DAPI colocalization (Fig. 7). UBE3A was also excluded from nuclear organelles such as nucleoli. Occasional gold/silver particles were associated with the nuclear membrane. In neuronal cytoplasm, UBE3A was seen decorating the Golgi apparatus and was associated with the outer mitochondrial membrane (Fig. 8c, d). In dendrites, labeling was often associated with the plasma membrane and with poorly defined tubulo-vesicular endomembranous structures. In dendritic spines, labeling was within the spine head and neck, including the spine apparatus, but was not found at the PSD (Fig. 8e). The majority of labeling within the neuropil was found in axon terminals making axospinous, axodendritic, and axosomatic synapses (Fig. 8e, f). In axon terminals, UBE3A associated with the plasma membrane and over clusters of presynaptic vesicles, but did not show a special affinity for the active zone. Double labeling experiments showed that UBE3A is expressed in GABAergic terminals (Fig. 8g).

## Discussion

While previous studies have examined human UBE3A expression using Western blot analysis [50, 51] and in human-induced pluripotent stem cells [52, 53], the present work provides the first high-resolution description of the subcellular distribution of UBE3A in the human brain. Though reporting only the presence of a molecule, not its functional level of activity, immunohistochemistry can shed light on important aspects of human neurophysiology and neuropathology inaccessible by even the most sensitive contemporary methods of live brain imaging. However, immunohistochemistry may be misleading unless tissue of excellent quality is studied. The potential for error is particularly troublesome when tissue of poor quality is examined using super-resolution methods. Several indirect methods are customarily used to assess the quality of human histological material including tissue pH and specific markers of RNA quality [54]. We used electron microscopy to assess tissue quality directly, finding that histological and immunocytochemical staining at the light microscopic level can appear to be satisfactory even in material that has been severely degraded, potentially resulting in misleading results.

Based on examination of surgically resected tissue of high ultrastructural quality, we conclude that UBE3A is broadly expressed by both excitatory (pyramidal) and inhibitory (GABAergic) neurons of the human cerebral cortex. Epilepsy, a common comorbidity in AS, is thought to reflect a disrupted balance between excitatory and inhibitory neurotransmission. We recently demonstrated in an animal model that selective deletion of *Ube3a* in GABAergic neurons causes both circuit hyperexcitability and seizures; the presence of UBE3A in inhibitory neurons of the human neocortex further supports the hypothesis that GABAergic neurons play a key role in precipitating the electroencephalographic abnormalities and epilepsy seen in AS.

UBE3A may also serve important functions in glia. For example, flies with glia-specific overexpression of Dube3a (the fly UBE3A homolog) display a robust seizure phenotype, suggesting a possible role for glial UBE3A in Dup15q pathophysiology [55]. Our finding that UBE3A is also expressed in glia in the human brain (though at a lower level than in neurons) is consistent with this possibility.

We found that UBE3A is expressed in multiple subcellular compartments. In neurons, it concentrates in euchromatin-rich domains within the nucleus, where it is positioned to regulate the expression of active genes, possibly exerting cell-wide global effects. A recent study shows that increasing UBE3A in the nucleus downregulates the glutamatergic synapse organizer Cbln1 [26], leading to weakened glutamatergic transmission; the same study found impaired sociability in these mutant mice, providing a possible explanation for how UBE3A overexpression might lead to autism spectrum disorder. Nuclear UBE3A may also perform additional roles. Like other ubiquitin E3 ligases, UBE3A may modulate the abundance and activity of transcriptional activators via its ubiquitin-protein ligase activity, thus modulating the formation of transcriptional complexes at genes and regulating chromatin structure [56]. UBE3A might also act more directly within the nucleus as a transcriptional co-regulator [36, 57–59].

UBE3A labeling was also associated with mitochondria. Loss of UBE3A has been linked to key enzymatic deficits in the mitochondrial respiratory chain [17, 18]. A role for mitochondrial abnormalities in the pathogenesis of autism spectrum disorders has also been suggested [60], and mitochondrial dysfunction has been shown in autistic patients with 15q inverted duplication [61].

The enrichment of UBE3A that we observed in both excitatory and GABAergic axon terminals and (to a lesser extent) in dendritic spines is of special interest since both Dup15q and AS mouse models show synaptic deficits [13, 25, 62]. Postsynaptically, abnormal function of excitatory synapses in UBE3A mutant mice has been linked to decreased UBE3A-mediated proteasomal degradation of the RhoA guanine exchange factor Ephexin5 and to pathways modulated by the synaptic protein Arc [23, 24, 58, 63]. UBE3A has also been shown to target the small-conductance potassium channel SK2 for degradation. Decreased NMDA receptor activation due to an elevated SK2 protein level may underlie the impaired LTP seen in AS model mice [62]. Presynaptically, the localization of UBE3A over vesicle clusters is surprising, since no integral synaptic vesicle proteins have yet been identified as targets for UBE3A [64]. However, UBE3A does appear to modulate the degradation of α-synuclein, a protein closely associated with synaptic vesicles [65–68], and loss of UBE3A can alter rates of synaptic depression [25, 48]. Although the mechanistic basis of UBE3A-associated synaptic disruption remains unclear, our data support a role for UBE3A in regulating synapses in the human neocortex.

The overall pattern of UBE3A immunoreactivity we found in human temporal cortex is generally consistent with that seen in previous studies of rodent cortex, reinforcing the idea that mice offer a valuable model in which to study the role of UBE3A in the brain. Mouse models of AS display many Angelman-like phenotypes, including learning and memory deficits, motor dysfunction, and seizures, while transgenic mice expressing UBE3A at increased dosages manifest several core autistic behavioral traits [26]. Nevertheless, some divergence between mouse and human UBE3A expression is likely. In particular, regulatory elements may have evolved in humans to sculpt UBE3A expression (which is spatiotemporally ubiquitous in the mouse) into unique and discrete patterns across the neocortex and throughout the cerebrum during development [69]. This remains to be tested in diverse samples of the developing and adult human brain.

## Conclusions

That UBE3A is expressed at high levels in both excitatory and inhibitory neurons in the human cerebral cortex points to the many ways that its dysregulation might disrupt circuit function, while its concentration at synapses and in nuclei offers intriguing functional clues. UBE3A in axon terminals presumably regulates the function of individual synapses; in contrast, its concentration in euchromatin-rich nuclear domains suggests that UBE3A may also mediate global effects on neuronal physiology, by regulating gene transcription. This information identifies axon terminals and nuclei as two distinct potential targets for future clinical intervention.

## Abbreviations

DMSO: Dimethyl sulfoxide
E6-AP: E6AP ubiquitin-protein ligase
GABA: γ-Aminobutyric acid
GFAP: Glial fibrillary acidic protein
HECT: Homologous to E6-AP carboxyl-terminus
NDBB: Neurodegenerative disease brain bank
Olig2: Oligodendrocyte transcription factor 2
PB: Phosphate buffer
PBS: Phosphate-buffered saline
PFA: Paraformaldehyde
RAD23A: Rad23 homolog A, nucleotide excision repair protein
RIN: RNA integrity number
UBE3A: Ubiquitin-protein ligase E3A
UPP: Ubiquitin-proteasome pathway
